# Anti-Angiogenic Drugs Inhibit Interstitial Lung Disease Progression in Patients With Advanced Non-Small Cell Lung Cancer

**DOI:** 10.3389/fonc.2022.873709

**Published:** 2022-06-20

**Authors:** Yanning Wang, Xiaoling Gong, Yuxuan Hu, Qing Yi, Qianning Zhang, Liyun Miao, Yujie Zhou

**Affiliations:** ^1^ Clinical Stem Cell Center, The Affiliated Drum Tower Hospital of Nanjing University Medical School, Nanjing, China; ^2^ Department of Pharmacy, Nanjing Drum Tower Hospital, The Affiliated Hospital of Nanjing University Medical School, Nanjing, China; ^3^ Institute of Pharmaceutical Sciences, China Pharmaceutical University, Nanjing, China; ^4^ Department of Respiratory and Critical Care Medicine, Nanjing Drum Tower Hospital, Nanjing, China; ^5^ Department of Respiratory and Critical Care Medicine, Nanjing Drum Tower Hospital Clinical College of Nanjing University of Chinese Medicine, Nanjing, China

**Keywords:** non-small cell lung cancer, interstitial lung disease, acute exacerbation, anti-angiogenic, chemotherapy

## Abstract

**Background:**

Interstitial lung disease (ILD) is the most serious complication of chemotherapy in lung cancer patients with pre-existing ILD. The effect of anti-angiogenic drugs in lung cancer patients with ILD remains unclear. We examined the effect of anti-angiogenic drugs on reducing the risk of ILD progression in non-small cell lung cancer (NSCLC) patients receiving chemotherapy.

**Methods:**

We analyzed the risk of ILD progression in 52 patients with advanced NSCLC with ILD who received first-line chemotherapy with (anti-angiogenic group, n = 22) and without (non-anti-angiogenic group, n = 30) anti-angiogenic drugs between August 2014 and January 2021.

**Results:**

The incidences of chemotherapy-related ILD progression were significantly lower in the anti-angiogenic than in the non-anti-angiogenic groups (0% vs. 20.0%, p = 0.033). However, there were no differences in other events as the competing risk factors of ILD progression between the two groups. The overall-cumulative incidence of ILD progression during the first-line and subsequent chemotherapy was 30.8% (16 of the 52). The median progression-free survival had no significant difference between the anti-angiogenic and the non-anti-angiogenic groups (10.3 vs. 8.1 months, p = 0.386).

**Conclusions:**

The addition of anti-angiogenic drugs to chemotherapy regimens may reduce the risk of chemotherapy-related ILD progression in patients with NSCLC-ILD.

## Background

In recent years, new treatments regimens for non-small cell lung cancer (NSCLC) have developed rapidly, such as targeted therapy and immunotherapy, which can significantly prolong the progression-free survival (PFS) and overall survival (OS) of patients. However, these treatment regimens can induce the occurrence of interstitial lung disease (ILD), and NSCLC patients with pneumonia have a higher incidence of ILD (1–3). In contrast, chemotherapy may be a more appropriate treatment option. However, our previous meta-analysis indicated that first-line chemotherapy may be associated with a higher rate of acute exacerbation of interstitial lung disease (AE-ILD), the pooled AE-ILD rate was 8.07% (95% CI: 6.12-10.26%) (4). Therefore, there is an urgent need for new strategies to treat such patients.

The development of anti-angiogenic drugs had brought new hope for the treatment of lung cancer patients. A variety of anti-angiogenesis drugs had been developed, including endostar, bevacizumab and apatinib, etc. These drugs were often used in combination with chemotherapy to play a synergistic effect and prolong the survival time of lung cancer patients (5). Among them, the anti-angiogenic drug nintedanib in combination with docetaxel has shown a survival benefit in the second-line treatment of patients with advanced lung adenocarcinoma (6). At the same time, nintedanib has also become a specific drug for the treatment of pulmonary fibrosis. Studies have shown that nintedanib can significantly delay the decline of lung function and improve the life quality of pulmonary fibrosis patients (7, 8). Therefore, anti-angiogenic drugs may have dual effects of anti-cancer and anti-fibrosis.

A recent study showed that first-line chemotherapy combined with bevacizumab can reduce the risk of chemotherapy-related AE-ILD in NSCLC-ILD patients (0% vs 22.6%, P=0.037) (9), so whether anti-angiogenic drugs can inhibit the ILD progression in NSCLC patients with pre-existing ILD is worthy of further exploration.

## Methods

### Patients

We reviewed retrospectively medical records of patients with advanced NSCLC and pre-existing ILD who received first-line chemotherapy at Nanjing Drum Tower Hospital between August 2014 and January 2021. We enrolled patients according to the following inclusion criteria: age ≥ 18 years, histological or cytological confirmation of advanced NSCLC, at least 2 cycles chemotherapy in the first-line treatment, diagnosis of ILD, performance status (PS) 0-1 and organ function is sufficient for chemotherapy. Patients who had ILD with known etiology, such as collagen vascular disease, pneumoconiosis and drug-induced pneumonia; had a history of radiotherapy and chemotherapy; had pre-existing histories of AE-ILD and had received antifibrotic agents, such as pirfenidone and nintedanib were excluded from the study.

### Definition of ILD and ILD Progression

Pre-existing ILD was diagnosed according to clinical characteristics and pretreatment chest high-resolution computed tomography (HRCT) findings. All patients underwent HRCT according to standard clinical practice, and the presence of ILD was evaluated by two physicians (LYM and QZ). ILD including idiopathic pulmonary fibrosis (IPF), nonspecific interstitial pneumonia (NSIP), desquamative interstitial pneumonitis (DIP) and respiratory bronchiolitis-associated interstitial lung disease (RB-ILD) was diagnosed according to the international consensus classification of the American Thoracic Society/European Respiratory Society (ATS/ERS) (10). CT findings of pre-existing ILD in our study were classified into two groups: usual interstitial pneumonia (UIP) and non-UIP. The HRCT features of UIP were as follows: the distribution of lesions is usually mainly in the lower lung and subpleura, with grid shadow and honeycomb shadow of the lungs, often accompanied by traction bronchiectasis; ground glass shadow is visible, but the lesion area is smaller than the grid film. When HRCT lacks the above signs, it is classified as non-UIP type (11).

Chemotherapy-related ILD progression was defined as newly developed bilateral ground-glass abnormality and/or consolidation superimposed on pretreatment interstitial shadows within 4 weeks after the last cycle of the first-line chemotherapy; serum lactate dehydrogenase, C-reactive protein, KL-6 or surfactant protein A or D was elevated; no evidence of pulmonary infection and no radiotherapy during the treatment. In addition, if dyspnoea worsens within 30 days, it was defined as chemotherapy-related AE-ILD.

### Outcomes

The primary endpoint for comparing the anti-angiogenic and non-anti-angiogenic groups was the cumulative incidence of ILD progression in the observation period. The observation period was defined as the time from the day of initiating first-line chemotherapy to 4 weeks after the end of first-line chemotherapy. Secondary endpoints were progression-free survival (PFS), calculated as the period from day 1 to the date of disease progression or death by any cause. If no disease progression or death occurred, the date of the last imaging examination was used as the study endpoint. The response to chemotherapy was assessed according to the Response Evaluation Criteria in Solid Tumors version 1.1.

### Data Collection and Statistical Analysis

All clinical and laboratory data were collected from patients’ medical records. We performed a descriptive analysis of the count data. The χ2 test was used for patient count data. Pearson χ2 test was used when all theoretical numbers ≥ 5, and Fisher’s exact test was used when any theoretical number < 5. The survival data PFS adopted the multiplicative limit method, namely the Kaplan-Meier method to estimate the median PFS and draw the survival curve. The Log-rank test was used to analyze the clinical characteristics of PFS by a single factor, and then based on the results of the single factor analysis, P<0.2 and factors considered clinically related to PFS were included in the Cox regression model for multivariate analysis, and the risk ratio (HR) and its 95% confidence interval (CI) were also given. All analyses were performed using R version 3.3.2, with p < 0.05 indicating statistical significance.

## Results

### Patient Characteristics

**Figure 1:**
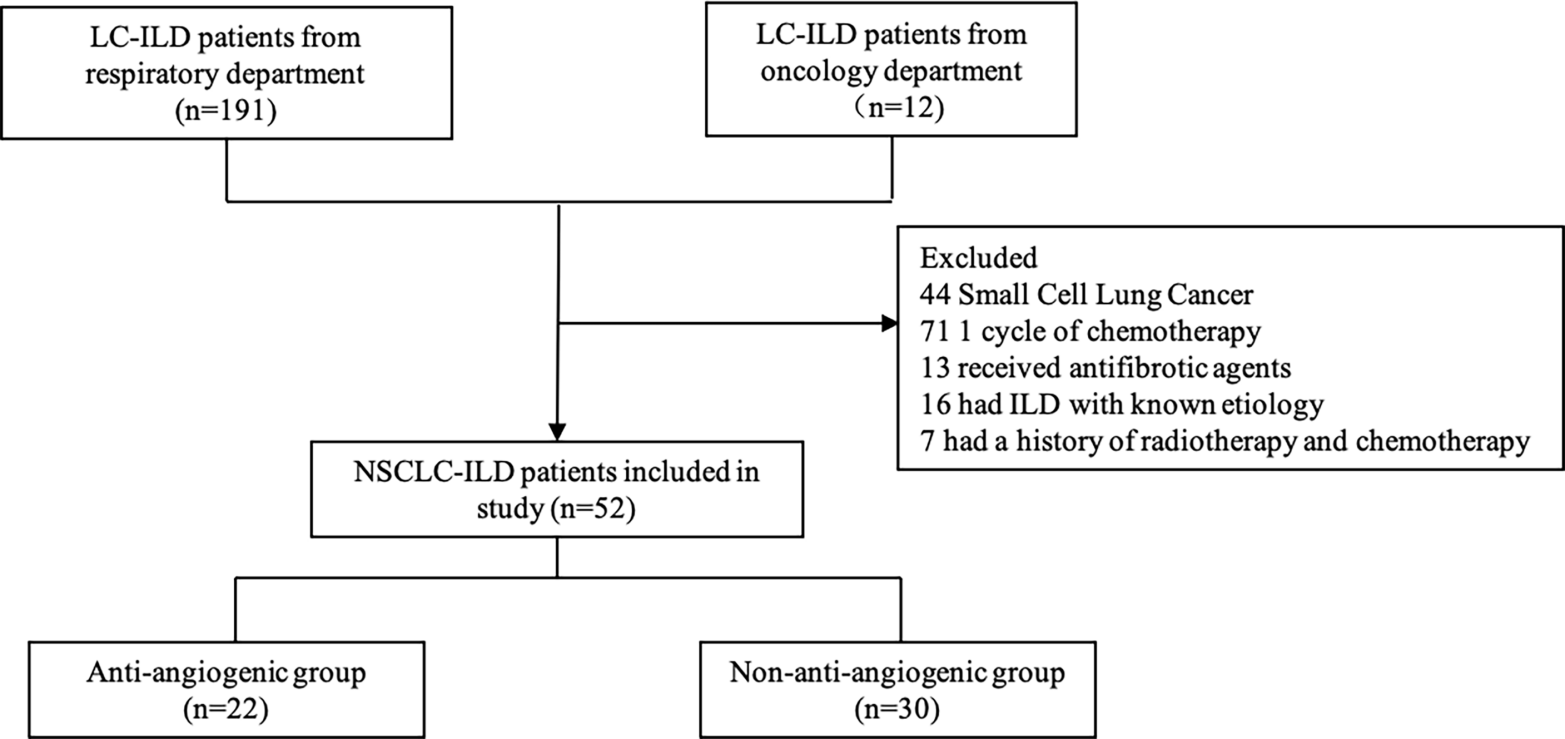
Flow Chart Diagram of Patients Selection.

We collected a total of 203 LC-ILD patients from the oncology department and respiratory department. 151 patients were excluded because they received radiotherapy and antifibrotic agents, and had ILD with known etiology. We finally enrolled 52 patients in this study and divided them into anti-angiogenic group and non-anti-angiogenic group (**Figure 1**).

Baseline characteristics were summarized in **Table 1**. Median age at the time of first-line chemotherapy was 67 years (IQR, 65.0-73.0), 16 of the 52 (30.8%) patients were never smokers. 29 of the 52 (55.8%) patients had adenocarcinoma. Stage III and IV diseases were observed in 19 (36.5%) and 33 (63.5%) patients, respectively. Most (67.3%) patients received chemotherapy more than 4 circles. Regarding the HRCT findings of ILD, most (84.6%) patients had a non-UIP pattern while the remainder had a UIP pattern. The average CT scan intervals were 6.6 weeks in anti-angiogenic group and 6.9 weeks in non-anti-angiogenic group. There were no significant differences between the anti-angiogenic and non-anti-angiogenic groups.

**Table 1:** Clinical Characteristics of 52 patients.

Clinical Characteristics	Total	Antiangiogenic group N (%)	Control Group N (%)	P Value
Patients	52(100)	22 (42.3)	30 (57.7)	
Gender				0.442
Male	44 (84.6)	20 (90.1)	24 (80)	
Female	8 (15.4)	2 (9.9)	6 (20)	
Age				0.399
Median	67	67	67.5	
Range	49-80	49-75	54-80	
<65	20 (38.5)	7 (31.8)	13 (43.3)	
>65	32 (61.5)	15 68.2)	17 (56.7)	
Smoke				0.640
Yes	36 (69.2)	16 (72.2)	20 (66.7)	
No	16 (30.8)	6 (27.3)	10 (33.3)	
Stage				0.575
III	19 (36.5)	9 (40.9)	10 (33.3)	
IV	33 (63.5)	13(59.1)	20 (66.7)	
Pathologic Types				0.473
Adenocarcinoma	29 (55.8)	11(50)	18 (60)	
Squamous Carcinoma	23 (44.2)	11(50)	12 (40)	
Classification of ILD				0.782
IPF	6 (11.5)	2 (9.1)	4 (13.3)	
Non-IPF	46 (88.5)	20 (90.9)	26 (86.7)	
ILD pattern				0.708
UIP Type	8 (15.4)	4(18.2)	4 (13.3)	
Non-UIP Type*	44 (84.6)	18(81.8)	26 (86.7)	
Cycle				0.190
<4	17 (32.7)	5(22.7)	12 (40)	
≥4	35 (67.3)	17(77.3)	18 (60)	
Regimens				0.176
AP	24 (46.2)	7 (31.8)	17 (56.7)	
GP	19 (36.5)	9 (40.9)	10 (33.3)	
TP	4 (7.7)	2 (9.1)	2 (6.7)	
PEM	5 (9.6)	4 (18.2)	1 (3.3)	

AP, pemetrexed+cisplatin; GP, gemcitabine+cisplatin; TP, nano albumin paclitaxel +platinum; PEM, pemetrexed.

*Non-IPF including nonspecific interstitial pneumonia (NSIP) (89%), desquamative interstitial pneumonitis (DIP) (4.5%) and respiratory bronchiolitis-associated interstitial lung disease (RB-ILD) (6.5%)

### First-Line Chemotherapy Regimens and Incidence of ILD Progression

First-line chemotherapy regimens are shown in **Table 1**. There is no significant difference between the anti-angiogenic and non-anti-angiogenic groups in the chemotherapy regimen (P=0.176). The most common regimen used for first-line chemotherapy in the anti-angiogenic group was platinum plus gemcitabine, and a total of 9 patients (40.9%) were enrolled. The most common regimen used for first-line chemotherapy in the non-anti-angiogenic group was platinum plus pemetrexed, and a total of 17 patients (56.7%) were enrolled. The risk of ILD progression after chemotherapy were 0% (0 of the 22 patients) and 20% (6 of the 30) in the anti-angiogenic and non-anti-angiogenic groups, respectively, and the difference in ILD progression rate was statistically significant (0% vs 20%, P=0.033; **Table 2**). Furthermore, in patients who received PEM-containing regimens, the risk of ILD progression had no significant difference in the anti-angiogenic and non-anti-angiogenic groups (0% vs 22.2%; P=0.268; **Table 2**).

**Table 2:** Incidence of ILD progression during frst-line chemotherapy.

Regimens	Antiangiogenic group		Control Group		P Value
	Number	ProgressN (%)	Number	ProgressN (%)	
AP	7	0 (0)	17	3 (17.7)	
GP	9	0 (0)	10	2 (20)	
TP	2	(0)	2	0 (0)	
PEM	4	0 (0)	1	1 (100)	
All	22	0 (0)	30	6 (20)	0.033
Including PEM scheme	11	0 (0)	18	4 (22.2)	0.268

AP, pemetrexed+cisplatin; GP, gemcitabine+cisplatin; TP, nano albumin paclitaxel+platinum; PEM, pemetrexed.

### Risk Factors of Chemotherapy-Related ILD Progression

We compared clinical parameters between 6 patients with and 46 without ILD progression during first-line chemotherapy to evaluate risk factors of ILD progression. Administration of anti-angiogenic drugs (p = 0.033) were significant (**Table 3**).

**Table 3:** Comparison of clinical factors between patients with and without ILD progression.

Clinical Characteristics	ILD progressN(%)	ILD non-progressN (%)	P Value
Patients	6	46	
Gender			0.573
Male	6	38	
Female	0	8	
Age			0.664
Median	67.5	67	
Range	54-75	49-80	
<65	3	17	
>65	3	29	
Smoke			0.160
Yes	6	30	
No	0	16	
Stage			1.00
III	2	17	
IV	4	29	
Pathologic Types			0.682
Adenocarcinoma	4	25	
Squamous Carcinoma	2	21	
Classification of ILD			0.540
IPF	1	5	
Non-IPF	5	41	
ILD pattern			0.573
UIP Type	0	8	
Non-UIP Type*	6	38	
Cycle			0.650
<4	1	16	
≥4	5	30	
Regimens			
Combination of anti-vascular drugs	0	22	0.033
Including pemetrexed	4	25	0.682

*Non-IPF including nonspecific interstitial pneumonia (NSIP) (89%), desquamative interstitial pneumonitis (DIP) (4.5%) and respiratory bronchiolitis-associated interstitial lung disease (RB-ILD) (6.5%).

### Comparison of Clinical Outcomes

**Figure 2:**
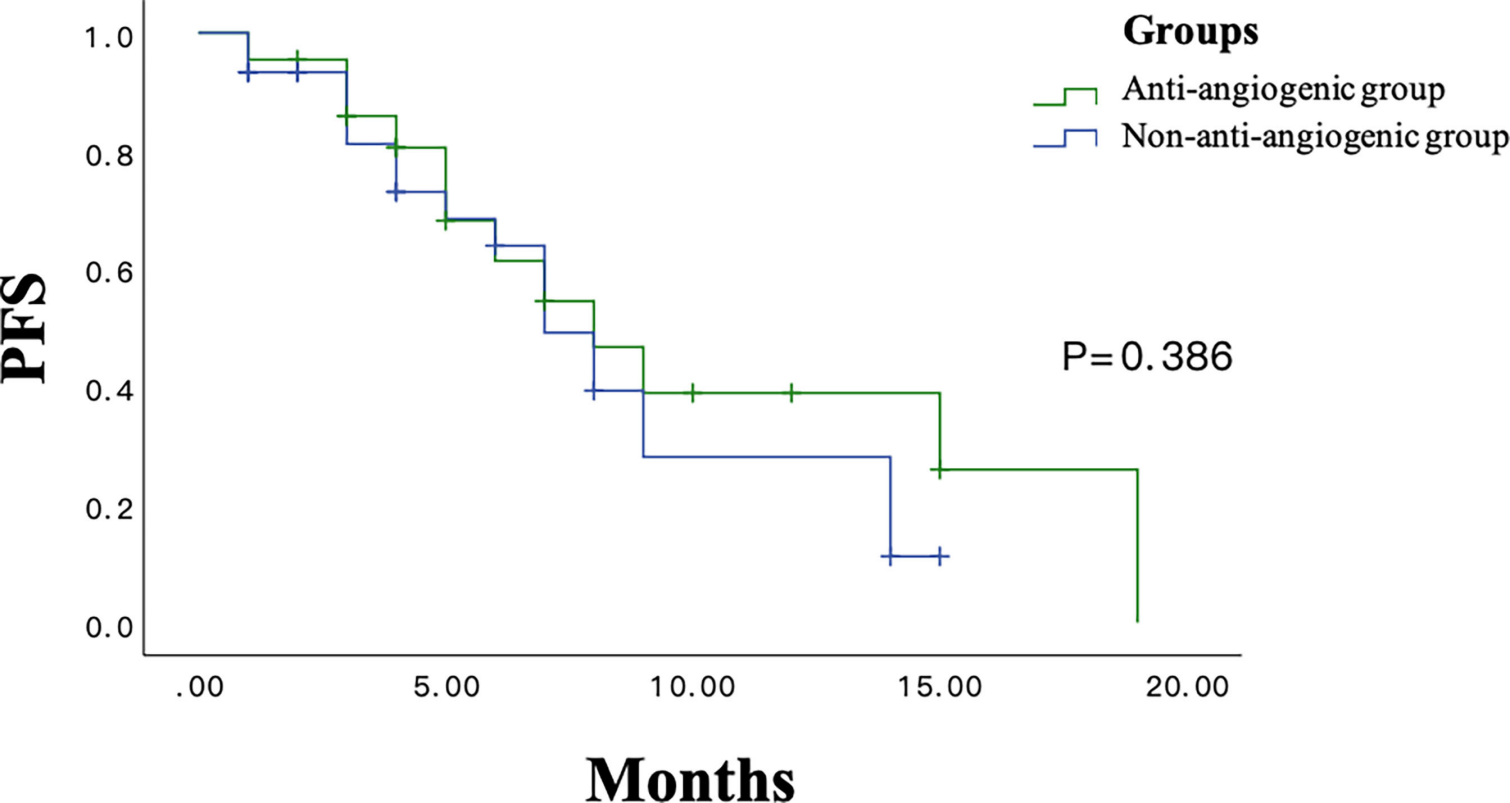
The PFS Survival Curve in Anti-angiogenic Groups and Non-anti-angiogenic Groups.

The survival curves in the anti-angiogenic and non-anti-angiogenic groups are shown in **Figure 2**. PFS had no significant differences between the anti-angiogenic group and the non-anti-angiogenic group (10.3 months; 95% confidence interval [CI], 7.0-13.5 vs 8.1 months; 95% CI, 6.3-9.9; p = 0.386; **Figure 2**). Overall response rate (ORR) and disease control rate (DCR) was not significantly different between the groups (ORR: 45.5% in the anti-angiogenic group vs 36.7% in the non-anti-angiogenic group, p = 0.523; DCR: 90.9% in the anti-angiogenic group vs 90% in the non-anti-angiogenic group, p = 1.00; **Table 4**).

**Table 4:** Comparison of the short-term efficacy and mPFS in test group and control group.

Curative effect	Antiangiogenic group	Control group	P value
CR	1	1	
PR	9	10	
SD	10	16	
PD	2	3	
ORR(%)	10 (45.5)	11 (36.7)	0.523
DCR(%)	20 (90.9)	27 (90)	1.00
mPFS (months)	10.3	8.1	0.386

## Discussion

Our retrospective study enrolled 52 NSCLC-ILD patients, including 22 patients in the antiangiogenic group and 30 patients in the control group. The rate of ILD progression related to first-line chemotherapy in the antiangiogenic group was significantly lower than that in the control group (0% vs 20%, P=0.033). Our results suggested that first-line chemotherapy combined with anti-angiogenic drugs can reduce the ILD progression rate for patients with NSCLC combined with pre-existing ILD.

Our result is consistent with the research conducted by Hamada et al. (9), which included a total of 48 patients with advanced NSCLC with ILD. They showed that the incidence of AE-ILD induced by first-line chemotherapy in the bevacizumab group was significantly lower than that in the non-bevacizumab group (0% vs 22.6%, P=0.037). In this study, most patients (83.3%) received pemetrexed-containing chemotherapy regimens, in this part of patients, the incidence of AE-ILD between the two groups also showed a significant difference (0% vs 24%, P=0.044). In our study, only 29 patients (55.7%) were treated with pemetrexed-containing regimens, the ILD progression related to first-line chemotherapy in the antiangiogenic group was lower than the control group (0% vs 22.2%). However, there was no significant difference (P=0.268) between two groups. Notably, the incidence of gemcitabine-induced ILD progression were higher than pemetrexed in our study (**Table 2**), this may explain why in only patients who received PEM-containing regimens, the incidence of ILD progression had no significant difference between two groups. Nevertheless, our results can also indicate that anti-angiogenic drugs can inhibit chemotherapy related ILD progression for NSCLC-ILD patients.

A meta-analysis conducted by Chen et al. (12) included 7 studies with a total of 251 patients. The incidence of AE-ILD related to first-line chemotherapy in NSCLC-ILD patients was 8.47%. Our updated meta-analysis included a total of 684 patients, and our results showed that the incidence of AE-ILD in NSCLC-ILD patients was 8.07%, similar to Chen’s study. The incidence of AE-IPF within 1 year in IPF patients is 3.6%-9.6% under natural progression (8), while chemotherapy-related AE-ILD mostly occurs within 4 months. Therefore, chemotherapy increases the incidence of AE-ILD. Previous studies had shown that the incidence of AE-ILD caused by various chemotherapy regimens was different. Two retrospective studies (13, 14) showed that the incidence of AE-ILD in NSCLC-ILD patients treated with pemetrexed-containing regimen was 12.5-22.6% (**Table S1**). In our study, the ILD progression rate in patients treated with pemetrexed-containing regimen was 17.7%, but no patients had AE-ILD, which may be contributed to the differences in patients’ baselines. A retrospective study analyzed 109 LC-ILD patients and found that patients with usual interstitial pneumonia (UIP) had a higher incidence of chemotherapy-related AE-ILD than non-UIP patients (30% vs 8%, P=0.005). Patients with UIP mode had higher AE-ILD mortality rate (15). In the study conducted by Hamada et al, UIP patients accounted for 50%, while UIP patients accounted for only 15.4% in our study, so it was hard to determine its impact on the ILD progression rate. Many studies (16–20) showed that the incidence of AE-ILD in patients adopt the platinum-containing albumin paclitaxel regimen was low, ranging from 0% to 8.3% (**Table S1**). At the same time, our meta-analysis showed that the incidence of AE-ILD in patients adopt this regimen was 4.98% (95%CI: 2.44-8.37%). Therefore, this regimen has the potential to become the most suitable chemotherapy regimen for patients with NSCLC-ILD. In our study, only 4 patients were treated with albumin paclitaxel and platinum-containing chemotherapy, and no patients developed ILD progression. Due to the insufficient sample size of patients enrolled in this regimen, we cannot perform subgroup analysis to verify whether this regimen has a lower ILD progression rate than other chemotherapy regimens. There is an ongoing phase II randomized controlled study (21), which aims to explore whether the nintedanib combined albumin paclitaxel and platinum-containing regimens prolong the interval to AE-IPF, and the results of this study are expected in the future.

In our study, the ORR and DCR had no significant difference between antiangiogenic group and control group (P>0.05). A study included 10 patients with NSCLC-ILD treated with chemotherapy combined with bevacizumab and 11 patients treated with chemotherapy alone (22). The ORR and DCR of the bevacizumab group was 40% and 90%, the ORR and DCR of the chemotherapy group was 27% and 82%. There was no significant difference between the two groups (P>0.05), which was consistent with our results. However, a phase III clinical trial (5, 23, 24) showed that anti-angiogenic drugs combined with chemotherapy can significantly increase the ORR of NSCLC patients (P<0.01). Our study did not show any short-term therapeutic benefit, which may be related to insufficient sample size and case selection bias.

In terms of the long-term efficacy of chemotherapy combined with anti-angiogenic drugs in the treatment of patients with advanced NSCLC, several clinical studies have shown that chemotherapy combined with anti-angiogenic drugs can significantly increase the PFS of patients with NSCLC compared to chemotherapy alone (25, 26). Up to now, a total of 3 studies have evaluated the long-term efficacy of chemotherapy combined with anti-angiogenic drugs in the treatment of NSCLC-ILD (9, 22, 27). The study conducted by Hamada et al. showed that the PFS of NSCLC-ILD in the bevacizumab group was significantly better than the chemotherapy group (8.0 months vs 4.3 months, P=0.026) (9). However, Shimizu et al. showed that the PFS of NSCLC-ILD patients in the bevacizumab group was similar with the chemotherapy group (5.5 months vs. 4.4 months, P>0.05) (22). In our study, the PFS of the antiangiogenic group and the control group was 10.3 and 8.1 months, respectively. There was no significant difference between the two groups (P>0.05), which did not show the value of long-term benefit. This may be related to insufficient sample size and publish bias.

### Significance and Limitations

To our knowledge, this is the first study to explore whether the application of anti-angiogenic drugs combined with chemotherapy can inhibit the ILD progression in NSCLC-ILD patients. The results of the study showed that anti-angiogenic drugs can reduce the progression rate of ILD in such patients, which provides new ideas for the first-line treatment of NSCLC-ILD. This study has several limitations. Firstly, this study was a small-scale retrospective study, giving rise to selection bias. Secondly, this study only includes Chinese patients. Some studies had shown that the incidence of chemotherapy-induced ILD was different in various ethnic groups (28, 29), so further research is needed to verify whether our results are equally applicable to other racial groups. Thirdly, the sample size of IPF and UIP patients in our study was small, so our findings were only applicable to non-IPF and non-UIP patients. Finally, studies had shown that ILD patients with poor basic lung function have higher incidence of AE-ILD and worse prognosis (27). Our study had a small sample size and lacked records of the basic lung function status, so it was hard to determine its impact on the ILD progression rate.

## Conclusion

This preliminary study suggests that anti-angiogenic drugs had lung protection and can reduce the risk of chemotherapy related ILD progression in NSCLC-ILD patients. Chemotherapy combined with anti-angiogenic drugs is a more appropriate treatment plan for first-line treatment of NSCLC-ILD patients. Further large-scale, randomized controlled studies are needed to confirm the effect of anti-angiogenic drugs on chemotherapy-related ILD progression and to develop better therapeutic managements for patients with lung cancer and pre-existing ILD.

## Data Availability Statement

The datasets presented in this study can be found in online repositories. The names of the repository/repositories and accession number(s) can be found in the article/**Supplementary Material**.

## Author Contributions

YW and XG contributed equally to this work. LM and YZ contributed equally to this work. All authors contributed to the article and approved the submitted version.

## Funding

Support for the study was provided by “Six Talent Peaks” of Jiangsu Provincial Department of Human Resources and Social Security grants 2014-WSN-047, Nanjing Medical Science and Technology Development Key Project grants ZKX15020, and Wu Jieping Foundation grants 320.6750.19081.

## Conflict of Interest

The authors declare that the research was conducted in the absence of any commercial or financial relationships that could be construed as a potential conflict of interest.

## Publisher’s Note

All claims expressed in this article are solely those of the authors and do not necessarily represent those of their affiliated organizations, or those of the publisher, the editors and the reviewers. Any product that may be evaluated in this article, or claim that may be made by its manufacturer, is not guaranteed or endorsed by the publisher.
