# Supplementary material for: Anti-Angiogenic Drugs Inhibit Interstitial Lung Disease Progression in Patients With Advanced Non-Small Cell Lung Cancer
Source: Front Oncol. 2022 Jun 20;12:873709. doi: 10.3389/fonc.2022.873709 (PMC9251331; doi:10.3389/fonc.2022.873709)
Supplement: Supplementary file 1 [file Table_1.docx]

**Supplementary material**

**Supplemental Table 1. Characteristics of studies related to NSCLC-ILD**

| **Authors, year** | **Study Design** | | **Treatment** | **Patients Enrolled** | **Mean Age,y** | **Male, n(%)** | **Current/Former Smokers, n(%)** | **Stage (n)** | **ILD Pattern (n)** | **PS (n)** |
| --- | --- | --- | --- | --- | --- | --- | --- | --- | --- | --- |
| Kenmotsu 2019 | phase2 | | CB+nab-PTX | 94 | 70 | 89 |  | IIIA/IIIB/IV/Recurrent  (15/23/47/9) | UIP/Non-UIP  (50/44) | 0/1  (42/52) |
| Asahina 2019 | phase2 | | CB+nab-PTX | 36 | 68.5 | 72.2 | 97.2 | IIIB/IV/Recurrent  (15/18/3) | UIP/Non-UIP  (12/24) | 0/1  (13/23) |
| Minegishi 2011 | pilot | | CB+weekly PTX | 18 | 71 | 77.8 | 83.3 | IIIA/IIIB/IV or Recurrent  (2/3/13) | UIP/Non-UIP  (6/12) | 0/1  (7/11) |
| Fukuizumi 2019 | phase2 | | CB+weekly PTX | 35 | 68 | 88.6 | 94.3 | IIIA/IIIB/IV/Recurrent  (15/7/10/3) | UIP/Non-UIP  (18/17) | 0/1  (18/17) |
| Cabiddu/Sekine  2016 | pilot | | CB+S-1 | 21 | 67 | 90.5 | 95.2 | IIB/IIIIA/IIIB/IV/ Recurrent  (1/2/10/4/4) | UIP/Non-UIP  (12/9) | 0/1/2  (7/12/2) |
| Hanibuchi 2018 | phase2 | | CB+S-1 | 33 | 70 | 90.9 | 90.9 | IIIB/IV/Recurrent  (7/19/7) | UIP/Non-UIP  (22/11) | 0/1  (18/15) |
| Yasuda 2018 | Retrospective | | CB+nab-PTX | 12 | 73 | 91.7 | 100 | IIIA/IIIB/IV  （1/4/7） | UIP/Non-UIP  （3/9） | 0-1/2  （11/1） |
| Watanabe 2013 | Retrospective | | CB+weekly PTX  CB+DOC  NVB | 21 | 68.4 | 85.7 | 100 | IIIB/IV  (11/10) | UIP/Non-UIP  (18/3) | 0/1/2  (8/10/3) |
| Watanabe 2015 | Retrospective | | DDP+NVB | 67 | 64 | 95.5 | 100 | IIIB/IV/Recurrent  (20/42/5) | UIP/Non-UIP | 0/1/2  (11/53/3) |
| Shukuya 2010 | Retrospective | | CB+weekly PTX | 15 | 68 | 86.7 |  | IIIA/IIIB/IV/Recurrent  (1/5/7/2) | UIP/Non-UIP  (4/11) | 0/1/2  (6/7/2) |
| Kinoshita 2012 | Retrospective | | CB+weekly PTX  DDP+NVB DDP+DOC | 22 | 70 | 95.5 | 100 | IIIA/IIIB/IV or Recurrent  (1/6/15) | UIP/Non-UIP | 0/1  (12/10) |
| Igawa 2018 | Retrospective | | CB+nab-PTX | 34 | 71 | 85 | 97 | IIIA/IIIB/IV or Recurrent  (2/2/30) | UIP/Non-UIP  (16/18) | 0-1/2-3  (32/2) |
| **Authors, year** | **Study Design** | | **Treatment** | **Patients Enrolled** | **Mean Age,y** | **Male, n(%)** | **Current/Former Smokers, n(%)** | **Stage (n)** | **ILD Pattern (n)** | **PS (n)** |
| Yamaguchi. 2017 | Retrospective | | DDP+VP-16 | 24 | 63 | 95.8 |  | IIIA/IIIB/IV  (5/6/13) | UIP/Non-UIP  (20/4) | 0/1/2  (14/8/2) |
| Kenmotsu. 2015 | Retrospective | | platinum-based chemotherapy | 104 | 67 | 91.3 | 100 | IIIA or IIIB/IV/Recurrent  (41/55/8) | UIP/Non-UIP  (70/34) | 0-1/2  (96/8) |
| Shimizu. 2014 | Retrospective | | CB+weekly PTX | 11 | 72 | 91 | 91 | IIIA/IIIB/IV  (2/2/7) | UIP/Non-UIP  (3/8) | 0 or 1 |
| Araya. 2019 | Retrospective | | CB+nab-PTX | 9 | 69 | 88.9 | 100 | IIIB/IV  (2/7) | UIP/Non-UIP  (5/4) | 0-1/2  (7/2) |
| Niwa. 2017 | Retrospective | | CB+nab-PTX | 9 | 67 | 100 | 100 | IIIA/IIIB/IV  (1/1/7) | UIP/Non-UIP  (6/3) | 0/1/2  (0/8/1) |
| Fujita. 2019 | Retrospective | | PT+PEM | 24 | 70 | 91.7 | 95.8 | IIIA or IIIB/IV or Recurrent  (8/16) | UIP/Non-UIP  (2/22) | 0-1/2  (22/2) |
| Fujita. 2018 | Retrospective | | CB+nab-PTX | 8 | 77 | 87.5 | 100 | IIIA/IIIB/IV  (5/1/2) | UIP/Non-UIP  (4/4) | 0/1  (3/5) |
| Kakiuchi. 2017 | Retrospective | | platinum-based chemotherapy | 35 | 72 | 93.2 | 98.6 |  |  |  |
| Choi. 2014 | | Retrospective | CB+GEM  CB+PEM | 52 | 67 | 86.5 | 86.5 | I/II/III/IV  (2/2/11/37) |  | 0-1/2  (47/5) |

Abbreviations: PS: performance status; CB: carboplatin; DDP: cisplatin; PT: platinum; nab-PTX: nano albumin paclitaxel; PTX: paclitaxel; S-1: tegafur-gimeracil-oteracil potassium; DOC: docetaxel; NVB: vinorelbine; VP-16: etoposide; PEM: pemetrexed; GEM: gemcitabine; PS: performance status; UIP: usual interstitial pneumonia.
